# Antimicrobial resistance within the one health lens: global drivers, mechanisms and public health policy gaps in low resource settings

**DOI:** 10.3389/fcimb.2026.1862774

**Published:** 2026-07-24

**Authors:** Nathan Mugenyi, Ronald Omolo Ouma, Mohamed Mustaf Ahmed, Don Eliseo Lucero-Prisno

**Affiliations:** 1 Department of Community Health, Faculty of Medicine, Mbarara University of Science and Technology, Mbarara, Uganda; 2 Department of Physiology, Faculty of Medicine, Mbarara University of Science and Technology, Mbarara, Uganda; 3 Faculty of Medicine and Health Sciences, SIMAD University, Mogadishu, Somalia; 4 Department of Global Health and Development, Faculty of Public Health and Policy, London School of Hygiene and Tropical Medicine, London, United Kingdom; 5 Office for Research, Extension and Innovations, Bukidnon State University, Malaybalay, Bukidnon, Philippines; 6 Research Office, Palompon Institute of Technology, Palompon, Leyte, Philippines

**Keywords:** AMR mechanisms, antibiotics, antimicrobial resistance, global public health, one health, silent pandemic

## Abstract

**Background:**

Antimicrobial resistance (AMR) poses a significant threat to global healthcare systems in terms of poor clinical outcomes, reduced quality of life and economic burdens. It is therefore imperative to comprehend the impact of all One Health domains, such as humans, livestock, agriculture and ecosystems in the fight against AMR. This narrative review therefore aims to highlight the resistance mechanisms, AMR global burden and its contributing factors. Additionally, this review identifies currently existing information gaps, monitoring parameters and global AMR mitigation strategies.

**Methods:**

A structured literature search was conducted across multiple electronic databases, including PubMed, EMBASE, ScienceDirect, the Cochrane Library, and Google Scholar to identify relevant peer-reviewed studies on antimicrobial resistance (AMR). The search included articles published in English between January 2013 and January 2025. Additional references were identified through manual screening of reference lists and relevant global reports from organizations such as the World Health Organization (WHO) and the Global Antimicrobial Resistance and Use Surveillance System (GLASS). Studies were eligible for inclusion if they addressed AMR in human health, animal health, agriculture, food systems and environmental settings within a One Health framework with particular emphasis on low- and middle-income countries (LMICs) as classified by the World Bank. Articles focusing on AMR mechanisms, transmission pathways, surveillance systems, AMS, environmental dissemination, and public health consequences were considered for inclusion.

**Results:**

The review narrative identified five major themes: global AMR burden, resistance mechanisms, cross-sectoral drivers, surveillance gaps, and stewardship responses. Globally, AMR was linked to 4.95 million deaths in 2019, with 3.57 million deaths directly attributable to drug-resistant infections. The major pathogens included *Escherichia coli*, *Klebsiella pneumoniae*, and *Staphylococcus aureus*, which exhibited alarming resistance to last-line antibiotics. The overuse of antibiotics in healthcare and agriculture, poor sanitation, unchecked use of biocides and heavy metals, and weak AMR surveillance were identified as the principal drivers. Data from the WHO’s Global Antimicrobial Resistance Surveillance System (GLASS) confirmed the escalation of resistance among priority pathogens.

**Conclusion:**

AMR is a multifaceted One Health challenge that requires urgent and integrated efforts to be mitigated. Strengthening integrated One Health surveillance, AMS, wastewater monitoring, and LMIC laboratory capacity should become immediate global policy priorities. Without immediate and sustained interventions, AMR threatens to reverse decades of medical progress and compromise the achievement of global health objectives. AMR is spreading globally due to irrational (un-judicious) antimicrobial use, cross-sectoral contamination, and the genetic exchange of resistance determinants among bacterial populations. Resistance gene transfer, particularly via horizontal mechanisms, is most pronounced where human, livestock, and environmental (One Health) interfaces overlap.

## Introduction

Antimicrobial resistance (AMR) is broadly recognized as one of the most serious threats to global public health and development, undermining decades of progress in infectious disease control and routine clinical care across human, animal, plant, and environmental domains (One Health). The World Health Organization (WHO) considers AMR one of the most serious public health challenges facing the world today. WHO further warns that unless urgent measures are taken, society could enter a “post-antibiotic era,” where routine infections and minor injuries may once again become deadly (Resistance I.C.G.o.A, 2019; World Health O, 2023). In 2019, 4.95 million deaths were associated with bacterial AMR, 1.27millions of which were directly attributable to drug-resistant infections, a burden that disproportionately affects low- and middle-income countries (LMICs) (Murray et al., 2022). Beyond the human toll, the World Bank projects that unchecked AMR could reduce global Gross Domestic Product (GDP) by up to 3.8% annually by 2050 and push tens of millions into poverty, highlighting AMR as both a health and macroeconomic crisis (The World B, 2024).

AMR is driven by a complex web of behaviors and exposures across several sectors. Misuse, underuse and overuse of antimicrobials in human healthcare remain pervasive, while non-human uses in food-animal production and crop systems continue to shape selection pressures and environmental dissemination of resistance. Recent global estimates indicate that human antibiotic consumption increased by approximately 16% between 2016 and 2023 (from 29.5 to 34.3 billion defined daily doses), with large geographic variation and persistent gaps in Antimicrobial Stewardship (AMS) (Klein et al., 2024). Addressing AMR thus demands an integrated One Health action as outlined in the Quadripartite (WHO/FAO/WOAH/UNEP) One Health Joint Plan of Action (2022–2026).

The pathogen landscape is continuously evolving. In 2024, the WHO released an updated Bacterial Priority Pathogens List (BPPL) to guide R&D and public health investments. The revision reaffirmed carbapenem-resistant *Acinetobacter baumannii* and carbapenem- and third-generation cephalosporin-resistant Enterobacterales as critical priorities and added rifampicin-resistant *Mycobacterium tuberculosis*. It also reclassified carbapenem-resistant *Pseudomonas aeruginosa* from critical to high priority, reflecting the shifting resistance patterns across regions (Organization W.H, 2024; Ukoaka et al., 2024).

Although global surveillance capacity is expanding, it remains uneven. The WHO’s Global AMR and Use Surveillance System (GLASS) now integrates AMR with antimicrobial use/consumption (AMU/AMC) data, enabling countries to track trends and benchmark stewardship (including via the AWaRe framework) (World Health O, 2025). The 2025 GLASS AMU report summarizes the progress and data contributions from 60 countries, underscoring the importance of surveillance in informing policy and practice. In the animal health sector, data compiled by the World Organization for Animal Health (WOAH) show encouraging signs: between 2020 and 2022, antimicrobials intended for use in animals declined by approximately 5% (from 102 to 97 mg per kg of animal biomass), although absolute volumes and practices still vary widely, especially in settings with rapid livestock expansion (Mugenyi et al., 2024; World Organisation for Animal H, 2024). These trends emphasize that AMR is a multifaceted One Health challenge shaped by antimicrobial access, quality, and stewardship; infection prevention and control (IPC); water, sanitation and hygiene; and surveillance quality. Despite the expanding body of AMR literature, limited synthesis exists integrating molecular resistance mechanisms with environmental dissemination pathways and cross-sectoral One Health drivers, particularly within LMIC settings where surveillance and stewardship systems remain weak. This narrative review therefore aims to synthesize current evidence on the epidemiology, mechanisms, transmission pathways, drivers, and policy responses associated with AMR within a One Health framework, with particular emphasis on LMICs.

## Methods

### Study identification and selection

A comprehensive structured literature search was conducted across PubMed, EMBASE, ScienceDirect, the Cochrane Library, and Google Scholar to identify relevant peer-reviewed studies and global reports addressing antimicrobial resistance (AMR) within a One Health framework. Additional records were identified through manual screening of reference lists and reports from international organizations, including the World Health Organization and the Global Antimicrobial Resistance and Use Surveillance System. The search included studies published in English between January 2013 and January 2025.

**Figure 1:**
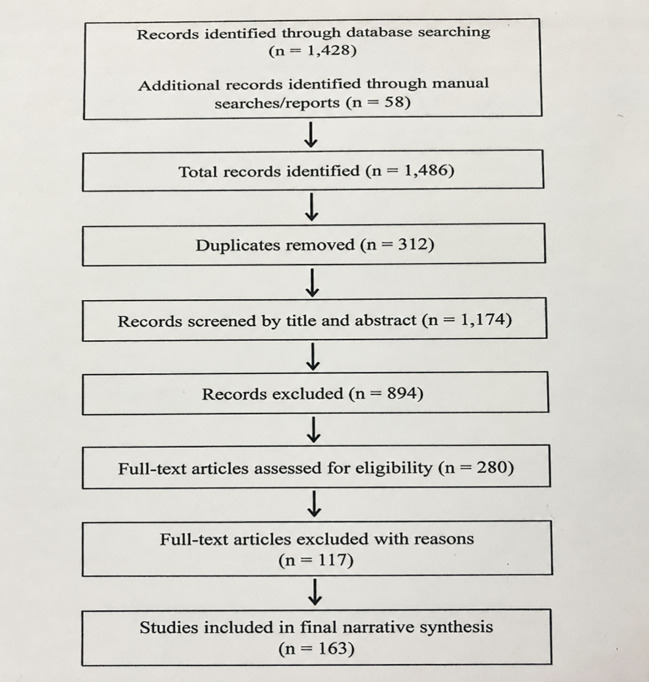
An illustration of the PRISMA-style study selection flow.

A total of 1,486 records were initially identified through database searches and manual screening. After removal of duplicates (n = 312), 1,174 records underwent title and abstract screening. Of these, 894 records were excluded due to irrelevance, non-One Health focus, non-human applicability, or lack of sufficient methodological detail. The remaining 280 full-text articles were assessed for eligibility. Following full-text review, 163 studies and global reports met the inclusion criteria and were included in the final narrative synthesis as shown in **Figure 1**.

### Search strategy

The search strategy combined controlled vocabulary terms and free-text keywords related to antimicrobial resistance, One Health, surveillance, and low- and middle-income countries (LMICs). Boolean operators (“AND”, “OR”) were used to optimize sensitivity and specificity across databases.

(“antimicrobial resistance” OR “AMR” OR “drug resistance”)

AND (“One Health” OR “environment” OR “livestock” OR “agriculture” OR “wastewater”)

AND (“LMICs” OR “low-income countries” OR “middle-income countries”)

AND (“surveillance” OR “stewardship” OR “transmission” OR “policy”).

### Quality appraisal and risk of bias assessment

Although this study was conducted as a narrative review, methodological quality and relevance of included studies were assessed narratively using predefined criteria including methodological clarity, consistency of findings, study relevance to One Health AMR transmission, and applicability to LMIC contexts. Where appropriate, elements of established appraisal frameworks, including the Critical Appraisal Skills Program (CASP) checklist and Joanna Briggs Institute (JBI) guidance, informed evaluation of study quality.

Studies with insufficient methodological transparency, unclear data sources, or limited relevance to the review objectives were excluded during full-text screening.

### Scope and depth of evidence synthesis

To improve analytical depth and global representativeness, this review synthesized evidence from more than 160 peer-reviewed articles, surveillance reports, and policy documents spanning human health, veterinary medicine, agriculture, environmental sciences, and public health policy. The expanded evidence base enabled broader thematic synthesis of AMR mechanisms, transmission pathways, environmental dissemination, surveillance systems, antimicrobial stewardship interventions, and emerging policy gaps across both LMIC and high-income settings.

## Results

This narrative review identified five major themes: global AMR burden, resistance mechanisms, cross-sectoral drivers, surveillance gaps, and stewardship responses.

Globally, AMR was linked to approximately 4.95 million deaths in 2019, with 3.57 million deaths directly attributable to drug-resistant infections (Murray et al., 2022). This mortality burden is higher than that of many major infectious diseases, underscoring the severity of the AMR crisis. Mechanistically, both intrinsic and acquired resistance have been widely reported in several studies. The most prevalent resistance mechanisms include enzymatic degradation of antimicrobials, such as β-lactamases, alteration of drug targets through mutations in DNA gyrase and ribosomal subunits, active efflux of antibiotics, and reduced membrane permeability. Horizontal gene transfer (HGT) through transformation, transduction, and conjugation is the primary pathway for the spread of antimicrobial resistance genes (ARGs).

These genetic exchanges are especially prevalent at the interface of human, animal, and environmental ecosystems (One Health), where microbial populations coexist and interact with each other. High resistance levels were consistently observed in Escherichia coli, Klebsiella pneumoniae, and Staphylococcus aureus, particularly against critical antibiotics such as carbapenems, colistin, and third-generation cephalosporins. These findings reflect the growing inefficacy of last-line treatments, especially in low-resource settings.

The major contributors to AMR include inappropriate prescribing and misuse (overuse and underuse) of antimicrobials in both clinical and agricultural settings, inadequate sanitation and wastewater management, widespread use of biocides and heavy metals, and the lack of robust AMR surveillance systems, particularly in LMICs. Data from the WHO’s GLASS further highlighted the rising resistance trends across key priority pathogens and antibiotic classes. These surveillance findings highlight the urgent need to strengthen AMR monitoring and response strategies to reduce preventable treatment failures and escalating healthcare costs.

## Discussion

### Emergence and historical context of AMR

Before World War II, the discovery and use of antibiotics marked a central moment in humanity’s ability to mitigate bacterial infections. Bacterial infections, which were once fatal, have become treatable and manageable. However, the often-quoted statement attributed to a U.S. general surgeon, claiming “It is time to close the book on infectious diseases and declare the war against pestilence won,” is likely an urban legend (Spellberg and Taylor-Blake, 2013). Since the dawn of antibiotics, scientists have acknowledged the emergence of AMR (Davies and Davies, 2010). When AMR first appeared as a public health concern in 1945, Sir Alexander Fleming issued a warning: “There is a danger that the ignorant man may easily underdose himself and by exposing his microbes to non-lethal quantities of the drug make them resistant” (Hudson et al., 2017).

Antimicrobials include antibiotics, which are prescribed for the treatment of bacterial diseases in humans and animals, and chemical biocides, which are employed in food processing environments for disinfection. The ability of bacteria to develop resistance to antibiotics through various mechanisms makes drugs less effective at killing or even inhibiting the growth of microorganisms. This resistance spreads over time, posing challenges for treatment and highlighting the need for proper antibiotic use, training, and continued research. Three resistance categories were explained. *In vitro* resistance, also known as microbiological resistance, is the lower limit of a bacterium’s susceptibility to antibiotics above a specified breakpoint. Microbiological resistance can be proven genotypically by utilizing molecular techniques to demonstrate the presence of a specific antimicrobial resistance gene or resistance mechanism. Furthermore, drug resistance exists.

This is based on the pharmacokinetic characteristics and typical susceptibility of the bacterial species. It is sensitive if the antibiotic’s minimal inhibitory concentration (MIC) for the relevant bacteria falls within the range of concentrations that the antimicrobial agent can reach. When the MIC of the antibiotic for the concentration of the bacteria in question is greater than what can be found at the infection site, the bacteria are said to be resistant. Lastly, clinical resistance, also known as *in vivo* resistance, indicates that a bacterial infection is no longer treatable and that treatment failure is apparent (Verraes et al., 2013). Research findings have demonstrated that a decrease in the biological fitness of bacteria can occasionally coexist with antibiotic resistance. This has been shown for Acinetobacter sp. resistant to rifampicin and Streptococcus pneumoniae resistant to macrolides (Kang and Park, 2010; Maher et al., 2012). Compared to susceptible pneumococci, antimicrobial resistance in pneumococci is followed by either unaltered or improved biological fitness, in contrast to other bacteria and resistance types (Rudolf et al., 2011).

### AMR as an emerging global public health concern

Research indicates that the global burden of AMR surpasses the 700,000 annual deaths estimated by the WHO and the United Nations (Organization W.H, 2018). AMR was once predicted to kill 10 million people annually by 2050 (Trotter et al., 2019); however, it has since been shown that the actual number is closer than previously believed. Globally, AMR developments vary greatly between nations and frequently confront distinct obstacles. AMR is a global issue that affects all countries, regardless of income. Although the development of new antibiotic classes has helped reduce infectious diseases, bacteria have quickly developed resistance. For example, resistance to streptomycin in patients with tuberculosis was reported just a year after its approval by the US FDA (Davies and Davies, 2010). When treating infections caused by bacteria that produce a broad range of β-lactamases or are multidrug-resistant, antibiotics such as carbapenems and colistin are often reserved as a last resort (Meletis, 2016). One of the WHO’s priority infections, Enterobacteriaceae, has become increasingly resistant to carbapenems and colistin worldwide (Du et al., 2016; Yang et al., 2016; Savin et al., 2020). Concerningly high resistance rates of approximately 83.35% were found in research for extensively drug-resistant (XDR) Klebsiella pneumoniae in newborn sepsis (Hassuna et al., 2020). Furthermore, multidrug resistance (MDR) was present in almost 500,000 new cases of rifampicin-resistant tuberculosis (RR-TB) detected globally in 2018 (Freitas and Werner, 2023).

The persistence of antibiotic tolerance can exacerbate medical conditions linked to diseases such as HIV, cancer, and malaria (Irfan et al., 2022). As the standard antibiotic treatment regimens become antiquated, the risk of AMR is expected to escalate sharply. This could potentially result in situations where patients who are terminally ill require palliative care, but the medications are no longer clinically effective (Ashiru-Oredope et al., 2013). Additionally, hospital intensive care units (ICUs) can become breeding grounds for nosocomial MDR pathogens. Finally, several studies have estimated that AMR-related diseases could cost USD 60–100 trillion by 2050, resulting in a 2% to 3.5% drop in the world total GDP (Sipahi, 2008; O'neill, 2014; Taylor et al., 2014). The ability of the human immune system to combat infectious infections is weakened by antibiotic resistance, which increases the risk of many problems in susceptible individuals undergoing dialysis, surgery, chemotherapy, and joint replacement (Control C.f.D. and Prevention, 2019). Furthermore, antibiotic resistance has a significant effect on individuals with long-term health issues, such as diabetes, asthma, and rheumatoid arthritis (Dadgostar, 2019). Since the persistence of AMR trends will reduce the effectiveness of antibiotics, healthcare workers should switch to last-resort medication classes, such as carbapenems and polymyxins, which are expensive, difficult to obtain in developing nations, and have a wide range of side effects (Organization W.H, 2015a).

### Antibiotics’ mechanism of action

Antibiotics are divided into ten main categories, most of which are based on naturally occurring compounds produced by bacteria and fungi, with a smaller portion being fully synthetic drugs created in the laboratory. Understanding how these drugs function is crucial for understanding resistance mechanisms (Walsh and Wencewicz, 2020). Antibiotics are grouped by their chemical structures and their action on bacteria in **Table 1**. They work by targeting key bacterial functions, such as blocking protein synthesis, disrupting cell wall formation, interfering with DNA replication, inhibiting folate production, or damaging the cell membrane (Crofts et al., 2017).

**Table 1:** Primary synthetic and natural antibiotic classes and their mode of action (Vikesland et al., 2019).

Antibiotic class	Description	Mode of action	Example
Synthetic			
Sulfonamides	Characterized by a sulfonamide group: R-S(=O)_2_-NH_2_. *R represents an Organic group	Locks the enzyme dihydropteroate synthase (DHPS) that is responsible for bacterial biosynthesis of folate.	Sulfamethoxazole, Sulfisoxazole, Sulfacetamide, Metrolazone.
Fluoroquinolones	Contain a fluorine substituent at Co of the bicyclic quinolone structure	Inhibit bacterial type II topoisomerases (DNA gyrase and topoisomerase (IV) required for bacterial DNA replication.	Ciprofloxacin, Levofloxacin, Ofloxacin.
Oxazolidinones	Completely synthetic antibiotic scaffolds	Lock bacterial protein synthesis by targeting the 50S ribosomal subunit.	Linezolid, Sutezolid, Eperezolid.
Natural			
beta-lactam- penicillins	A four-membered beta-lactam fused to a five membered saturated ring containing a sulfur atom.	Interfere with extracellular cross- linking and reduce the mechanical strength of the peptidoglycan layer.	Penicillin G
beta-lactam – cephalosporins	beta-lactam structure fused to a 4,6-bicyclic framework.	Interferes with extracellular cross- linking and reduce the mechanical strength of the peptidoglycan layer.	Cephalosporin C
beta-lactam – carbapenams	Carbon replaces the sulfur atom within the five membered ring of penicillin	Interferes with extracellular cross-linking and reduce the mechanical strength of peptidoglycan layer.	Meropenem
Aminoglycosides	Based upon an amino- ‘modified’ glycoside backbone.	Amino groups are positively charged at physiological pH and electrostatically interact with the negatively charged phosphate ion backbone of 16s rRNA in the small 30s ribosomal subunit.	Kanamycin A
Macrolactone polyketides (macrolides)	A ring with >8 atoms is connected by a lactone (oxo ester) bridge.	Targets the 50s ribosomal subunit.	Erythromycin A
Tetracyclines	Four-fused six- ‘member rings	Interact with specific sites on the 30s ribosomal subunit and inhibit bacterial protein synthesis	Tetracycline
Glycopeptides	A heptapeptide backbone that is cross- linked to produce a rigid, cup-shaped molecule.	Block cell wall assembly in susceptible bacteria	Vancomycin
Lipopeptides	consist of a lariat/lasso peptide structure	Disrupt outer cell membrane integrity and block protein biosynthesis	Colistin
Rifamyacins	A polyketide with a long bridge along one face of the cyclic scaffold.	Inhibits bacterial RNA polymerases thus imbedding the transcription of DNA to RNA.	Rifamyacin SV

The Global Antimicrobial Resistance Surveillance System (GLASS), an essential component of the Global Action Plan (GAP) against AMR, was introduced by the WHO in October 2015. Eight pathogens are now included in the surveillance system such as Neisseria gonorrhoeae, Salmonella spp., Shigella spp., Acinetobacter baumannii, Staphylococcus aureus, Streptococcus pneumoniae, Escherichia coli and Klebsiella pneumoniae (Organization W.H, 2015b). Penicillins, third- and fourth-generation cephalosporins (3GC and 4GC), carbapenems, fluoroquinolones, aminoglycosides, tetracyclines, polymyxins, macrolides, and co-trimoxazole are important classes of medications for antimicrobial susceptibility testing (AST).

AMR mechanisms can be broadly categorized as follows.

Intrinsic Resistance: Certain bacterial genera or species have unique structural or functional characteristics that provide resistance to specific antibiotics. These bacterial groupings are typically unsuccessful because they lack the target sites of antibiotics. Mycoplasma species resist glycopeptides and β-lactam antibiotics due to the absence of a cell wall. Some bacteria, such as *E. coli*, can produce enzymes (such as AmpC β-lactamase) to inactivate antibiotics or use export systems, such as AcrAB-TolC, to remove them (Schwarz et al., 2005).

Acquired resistance: When genetic codes from other bacterial strains are transferred to naturally susceptible bacteria, the bacteria become resistant to specific antibiotics. Three main processes account for acquired resistance (Schwarz et al., 2005).

### Evolution mechanisms and spread of AMR

Microbes have developed defensive mechanisms against antimicrobial compounds through Darwinian selection, such as blocking drug entry, producing degradation enzymes, and altering target (active site) configurations. Most antimicrobial drugs are modifications of naturally occurring compounds. According to metagenomic research, only a small fraction of the antibiotic resistance in soil microbes is linked to human diseases (Forsberg et al., 2014). Resistance to β-lactam antimicrobial medications is an example of a naturally occurring resistance mechanism that affects human health. These enzymes, known as β-lactamases, have been inactivating these antibacterial compounds for millions of years (Aminov, 2009). Earlier beliefs that antimicrobial compounds produced by saprophytic organisms inhibit neighboring bacteria have been revised. Recent research has shown that soil concentrations of these compounds are usually too low to fully prevent bacterial growth (Martinez, 2009).

Second, the data points to the possibility that antimicrobials, even at sublethal concentrations, have a significant impact on bacterial physiology. This could accelerate the rate at which microbes evolve by adaptive means and potentially serve as signaling molecules that affect the expression of host and microbial genes (Andersson and Hughes, 2014). Some saprophytic bacteria that produce carbapenems, a significant family of broad-spectrum antibiotics used in medicine, are particularly noteworthy in this regard. The genes linked to the production of carbapenems may also be involved in the creation of biofilms or the quorum-sensing apparatus, which is the system by which a colony of microorganisms regulates its growth and gene expression (Aminov, 2009). This procedure raises concerns regarding the unintentional consequences of antimicrobial medications, and our knowledge of their possible impact on bacteria beyond their inhibitory function remains limited (Morita et al., 2014).

### Resistance mechanisms to antimicrobial agents

Bacteria can develop resistance through various mechanisms, including enzymatic degradation of the drug, mutations that alter the antibiotic target, changes in cell wall permeability to reduce drug uptake, and the use of alternative metabolic pathways to bypass the antibiotic’s effects. One prevalent mechanism of antibiotic resistance is enzymatic breakdown and alteration. β-lactamase enzymes hydrolyze the β-lactam ring of β-lactam antibiotics, including cephalosporins, which are primarily harmful to gram-negative bacteria (Livermore and Woodford, 2006). Aminoglycosides are another class of antibiotics, and resistance to them is mostly mediated by enzymatic degradation; phosphotransferases, acetyltransferases, and nucleotide transferases are responsible for inactivating these antibiotics (Wright, 1999). There are numerous variations of each of these enzymes, and each has a distinct range for one or more antibiotics.

Target modification involves changing the target of the antibiotic, usually an enzyme, to prevent antibiotic binding and activity. This resistance mechanism is exemplified by mutations in gyrase and topoisomerase IV genes, which reduce the effectiveness of quinolones and fluoroquinolones (Drlica and Zhao, 1997). One type of horizontal transmissible target alteration is methicillin-resistant Staphylococcus aureus (MRSA). The mecA gene, which encodes a variant penicillin-binding protein PBP2A with a very low affinity for β-lactams, is present in MRSA. Low-affinity PBP2A is the only PBP that continues to function in the presence of β-lactams (Pinho et al., 2001). Altering the permeability of the bacterial cell wall or envelope can limit antibiotic entry or promote antibiotic efflux, thereby controlling the internal antibiotic concentration. Tetracycline resistance is an example in which acquired genes enhance the efflux of antibiotics from bacterial cells (McMurry et al., 1980). Overexpression of bacterial efflux pumps can cause multidrug resistance; however, this non-transferable mechanism has limited and unclear clinical significance. Finally, cells can develop resistance by adding a step that differs from the typical physiological process. This is typically caused by excess enzymes. This is demonstrated by the synthesis of an extra dihydrofolate reductase in Escherichia coli and Citrobacter sp., which binds to different anti-folate chemicals than the chromosomal enzyme and has trimethoprim resistance, as assessed by the R-plasmid (Pattishall et al., 1977).

### Antibiotic resistance through the spread of resistance genes

**Figure 2:**
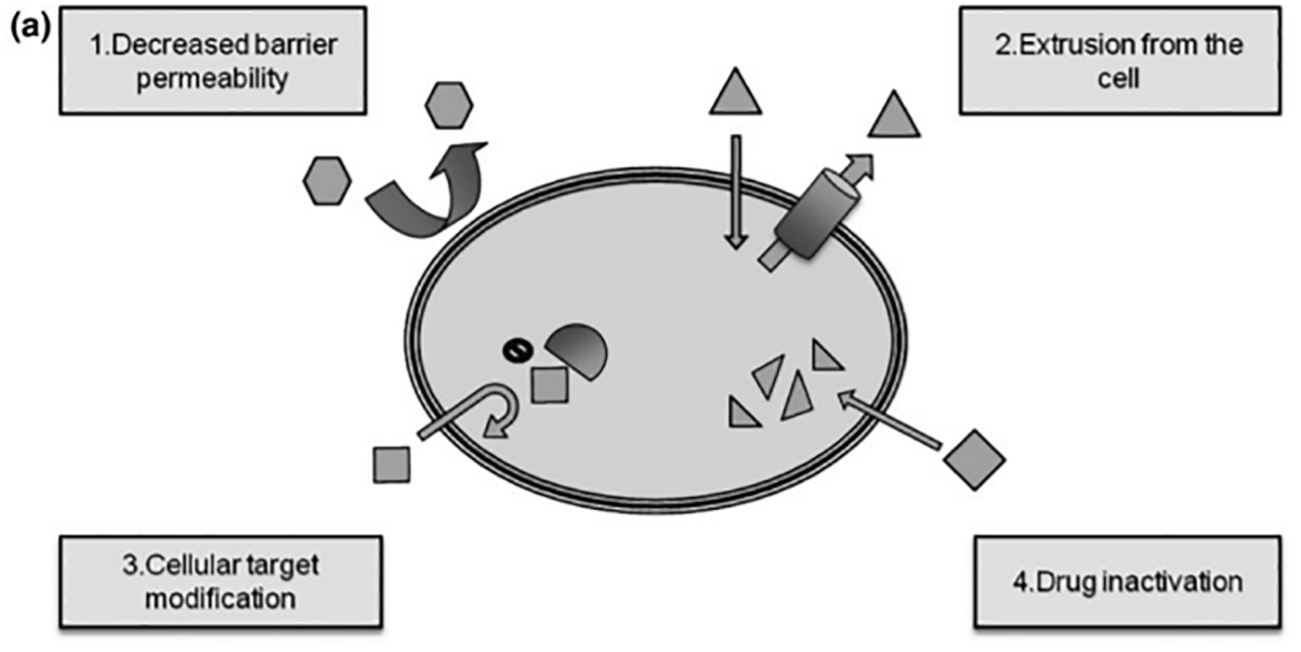
Complex AMR mechanisms (1) The primary mechanism of resistance is a decrease in membrane per4meability to the drug. (2) The medication actively pushes itself out of the cell. (3) Alteration of the cellular target of a medication. (4) Inactivation of the medication (Vittecoq et al., 2016).

Vertical gene transfer (VGT) and horizontal gene transfer (HGT) are key mechanisms driving the emergence and spread of AMR in bacteria. VGT involves the inheritance of resistance traits from parents to offspring, allowing resistant bacterial populations, such as those resistant to fluoroquinolones and oxazolidinones, to persist and expand over generations (**Figure 2A** (Silva et al., 2013; Caniça et al., 2015; Munita and Arias, 2016). The second pathway, also known as HGT (Husnik and McCutcheon, 2018), allows the interchange of genetic processes that promote resistance between different bacterial species (**Figure 2B**). Typically, HGT occurs through one of the following three mechanisms:

1. Transformation: This involves the introduction of exogenous DNA from the environment through the cell membrane. Both *in vitro* and in the environment, other cells take up free or “naked,” DNA transformation. A complex procedure is required for natural DNA transformation, involving type II and type IV secretion systems (T2SS and T4SS) as well as type IV pili, which allow bacteria to take up DNA from the surface and transfer it into the cytoplasm via a highly preserved channel in the cytoplasmic membrane (Krüger and Stingl, 2011).
2. Transduction: This is the transfer of genes from one bacterium to another via a viral medium. To transmit DNA between bacteria, such as Staphylococcus aureus, a bacteriophage or virus is needed (Coleman et al., 2006; Novick et al., 2010). Bacteriophages use bacterial cells for replication. One of the bacteriophages may become infected with bits of bacterial DNA (resistance sequence) during this process. To facilitate further replication, the carrying bacteriophage injects its DNA into a different bacterium. As a result, the resistance sequence can recombine with the DNA of the bacteria, and resistance is developed.
3. Conjugation: This is the direct transfer of genes from a donor cell to a recipient cell via plasmid-mediated direct cell-to-cell contact. Using T4SSs (this transferosome), genes are transported horizontally to recipient cells using this process (Juhas et al., 2008; Alvarez-Martinez and Christie, 2009). Furthermore, a coupling protein is needed to connect the two complexes at the start of conjugation, which is a DNA-binding complex at the DNA transfer site called the relaxosome. This leads to the donor and recipient cells sharing conjugative elements (such plasmids and transposons) (Catry et al., 2003; Dostál et al., 2011; Wong et al., 2012; Husnik and McCutcheon, 2018).

Transduction and transformation typically occur between closely related microbes. In contrast, conjugation between distinct phyla may result in the promiscuous spread of AMR. Plasmids are the most significant means of dispersing antibiotic-resistant genes (ARGs). These circular DNA constructs, known as plasmids, frequently serve as scaffolds for ARGs and mobile genetic elements (MGEs), such as integrons, transposons, and insertion sequences, which promote the growth of drug-resistant bacteria (MDR) (Hotopp, 2011; Zhu et al., 2013; Von Wintersdorff et al., 2016; Moser et al., 2018).

### Factors leading to the AMR epidemic

**Figure 3:**
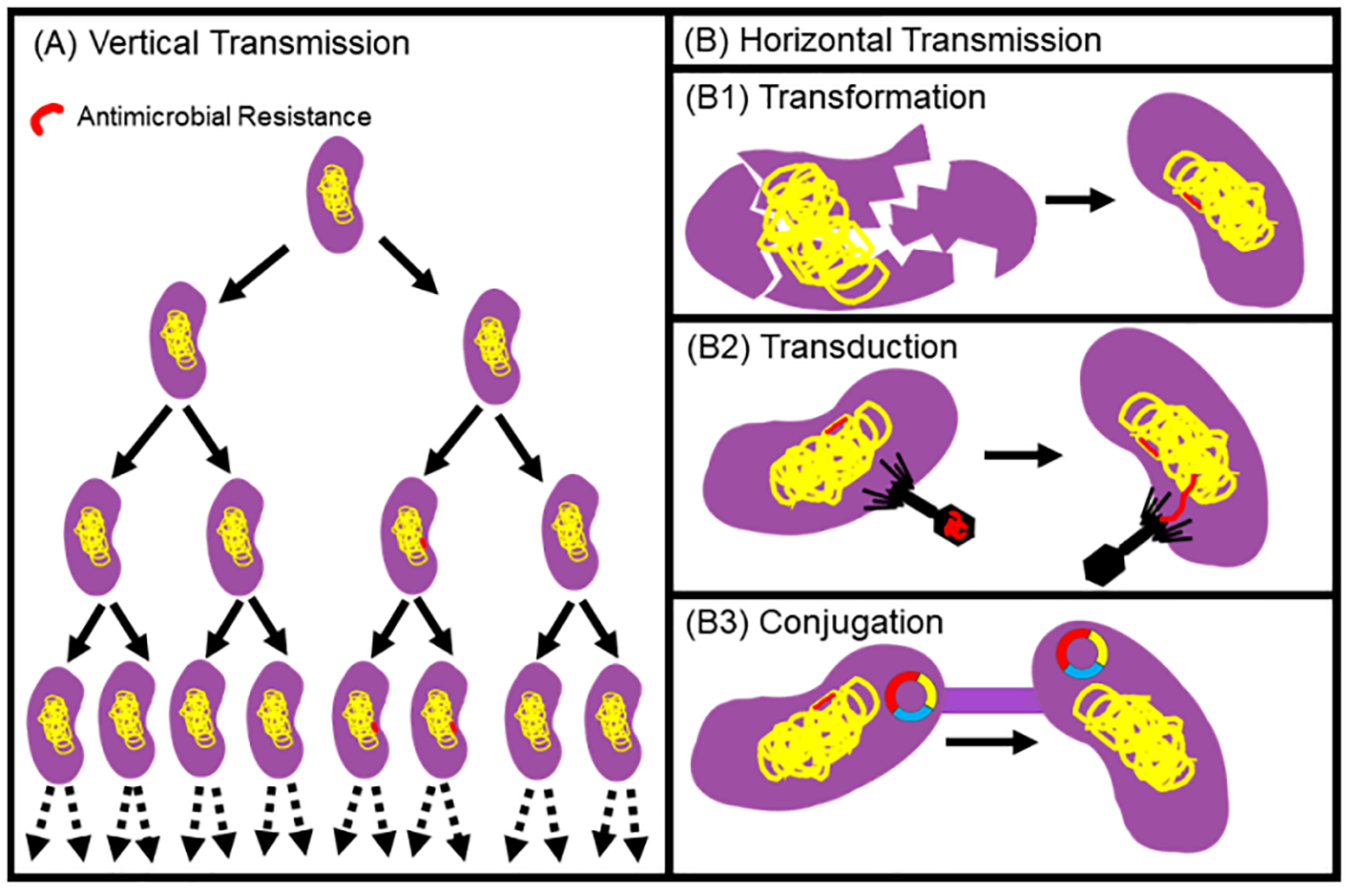
Primary pathways involved in the exchange of genetic information conferring antibiotic resistance consisting of **(A)** vertical transmission and **(B)** horizontal transmission (Hedman et al., 2020).

AMR is driven by complex cross-sectoral factors (Mudenda et al., 2025; Tadesse et al., 2017). Humans and animals share environments and diseases, some of which originate in animals (Zinsstag, 2012; Richardson et al., 2016; Collignon and McEwen, 2019). Resistant bacteria can spread across borders through direct contact, food, and the environment (Holmes et al., 2016; Richardson et al., 2016). AMR genes and organisms are found in humans, animals, food, plants, and environmental sources (Freitas and Werner, 2023). Population growth and dietary shifts, along with poor sanitation and pollution from hospitals and pharmaceutical waste, contribute to AMR. AMR is projected to cause 10 million deaths and cost the global economy USD 100 trillion by 2050 (O'neill, 2014; Trotter et al., 2019). AMR is emerging at an alarming rate owing to the abuse and misuse of currently available antimicrobials (**Figure 3**).

### Overuse of antibiotics

Global antibiotic use increased by 65% between 2000 and 2015 (Klein et al., 2018), with significant increases in countries such as China, Brazil, and Saudi Arabia (Irfan et al., 2022). Overuse and misuse are common in healthcare settings, especially in treating children, acute care, and conditions such as sinusitis and bronchitis (Milani et al., 2019; Schwartz et al., 2020). Studies have shown high rates of inappropriate antibiotic use in countries such as South Africa (Gasson et al., 2018), Pakistan (Sarwar et al., 2018), China (Wang et al., 2014), and Canada (Schwartz et al., 2020). This misuse reduces treatment effectiveness and fuels AMR (Davies and Bennett, 2017; Kariyawasam et al., 2022). During COVID-19, antibiotic over-prescription further contributed to AMR. In agriculture (Feeds, C.t.S.t.H.H.E.o.S.A.U.i.A, 1980), the rising global demand for meat and dairy products has driven the use of antibiotics for growth promotion and disease prevention. In 2017, veterinary antibiotic use (Grossman, 2014) reached 85,330 tons, and it is projected to increase by 11.5% by 2030 (Tiseo et al., 2020). Although regions such as the EU (Regulation, O, 2003) and the U.S. have introduced bans and regulations, 26 countries still used antibiotics as growth promoters in 2019 (Góchez et al., 2019).

### Biocides

Biocides are widely used in healthcare, agriculture, industry, and domestic settings for antimicrobial purposes (Kahrilas et al., 2015). Common biocides include ethanol, formaldehyde, chlorhexidine, and quaternary ammonium compounds (QACs), such as alkyldimethyl-benzyl ammonium chloride (ADBAC) and triclosan (Buffet-Bataillon et al., 2012). Unlike antibiotics, biocides often target multiple sites within or on bacteria, such as membranes and cellular components, and their effectiveness depends on concentration and environmental conditions (for example, pH, temperature, and organic matter) (Russell, 1995). Resistance to biocides can develop through mutations or acquired genes, similar to antibiotic resistance. Some bacteria, including Pseudomonas aeruginosa and *E. coli* (Irfan et al., 2022), have shown resistance to widely used disinfectants, such as chlorhexidine and silver compounds. Resistance genes (e.g., qacA/B) are often plasmid-borne and may also confer resistance to metals and antibiotics. Wastewater treatment plants are major release points for biocides into the environment, potentially contributing to the emergence of resistant microorganisms. In the EU, biocide use is regulated under the Biocidal Products Regulation (EU) 528/2012 (Irfan et al., 2022).

### Heavy metals

There are similarities between the antibiotic and metal resistance mechanisms in bacteria (Hobman and Crossman, 2015; Pal et al., 2017). Metals such as arsenic, zinc, cobalt, manganese, and silver utilize resistance strategies such as reduced membrane permeability, metal modification, efflux systems, target site changes, and metal sequestration (Baker-Austin et al., 2006), mechanisms that parallel those used against antibiotics such as β-lactams, tetracycline, and chloramphenicol. According to BacMe database analyses, copper resistance genes are among the most prevalent (Pal et al., 2014). The mobility and toxicity of metals in soil, water, and sediments are well documented (Pal et al., 2014), and the combined environmental exposure to antibiotics, metals, and biocides can surpass the minimum selection concentration (MSC), promoting resistance. Importantly, bacteria with metal resistance genes tend to also carry antimicrobial resistance genes (ARGs), often more frequently than those without metal resistance. This highlights the need to reassess environmental thresholds, particularly for practices such as sludge and manure application to land (Di Cesare et al., 2016).

### Antibiotic-associated environmental transmission networks

#### Drainage

A substantial quantity of active pharmaceuticals is excreted and enters wastewater systems, where it may be partially degraded, bound to sludge, or even remain in the effluent. Environmental studies have detected antibiotics and resistance genes in aquatic and sediment environments, such as fishponds and riverbeds. Oxytetracycline, in particular, accumulates significantly in sludge, while the partial removal of drugs such as ciprofloxacin and sulfonamides suggests differing environmental risks between sludge land application and effluent discharge into waterways (Irfan et al., 2022).

#### Veterinary and livestock

**Figure 4:**
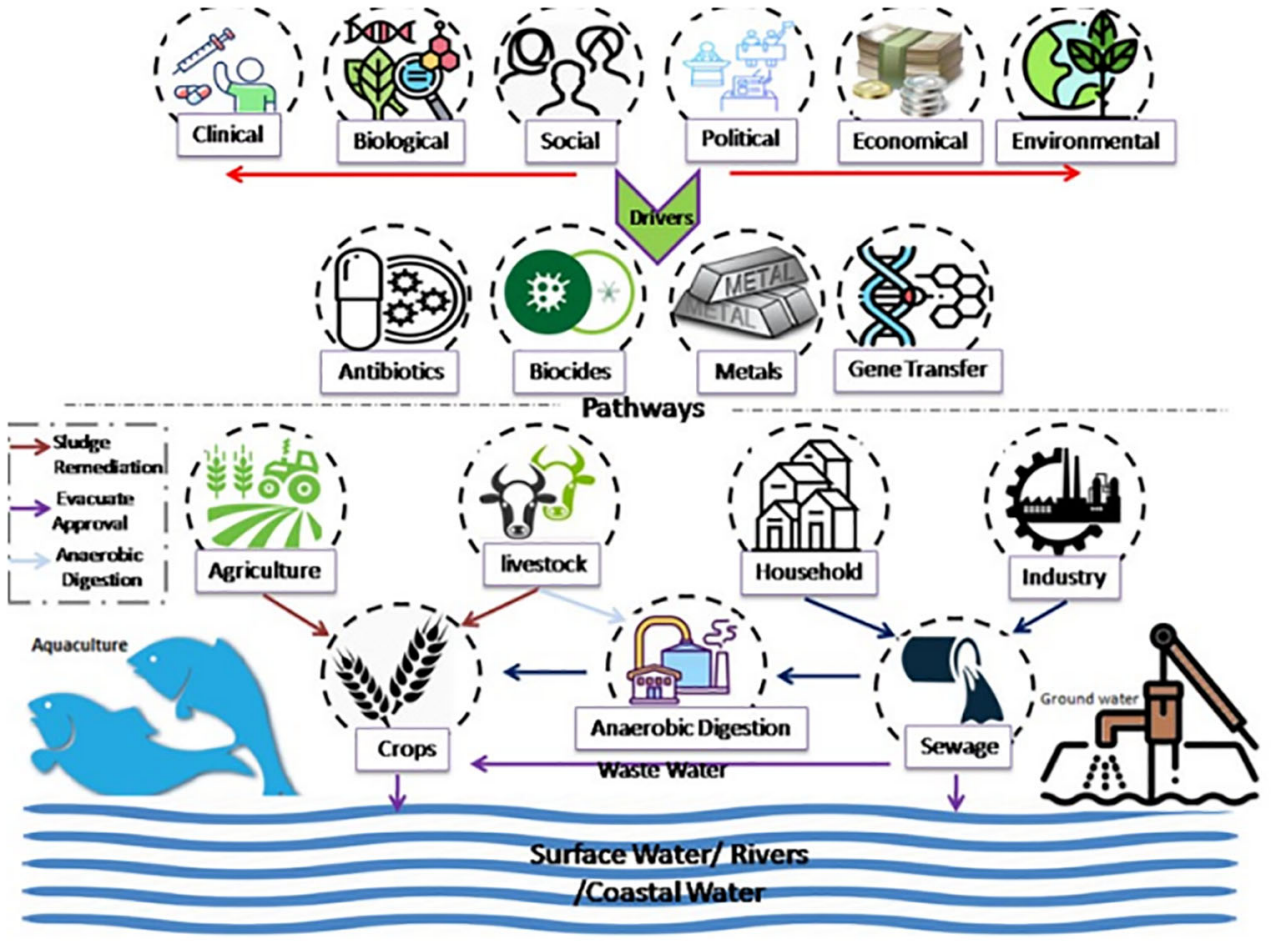
AMR is a global problem that impacts not only humans but also animals and ecosystems, both domestic and non-domestic. It is fueled by clinical, biological, social, political, economic, and environmental factors. The presence of AMR bacteria in the environment is a consequence of all these drives (Irfan et al., 2022).

**Figure 5:**
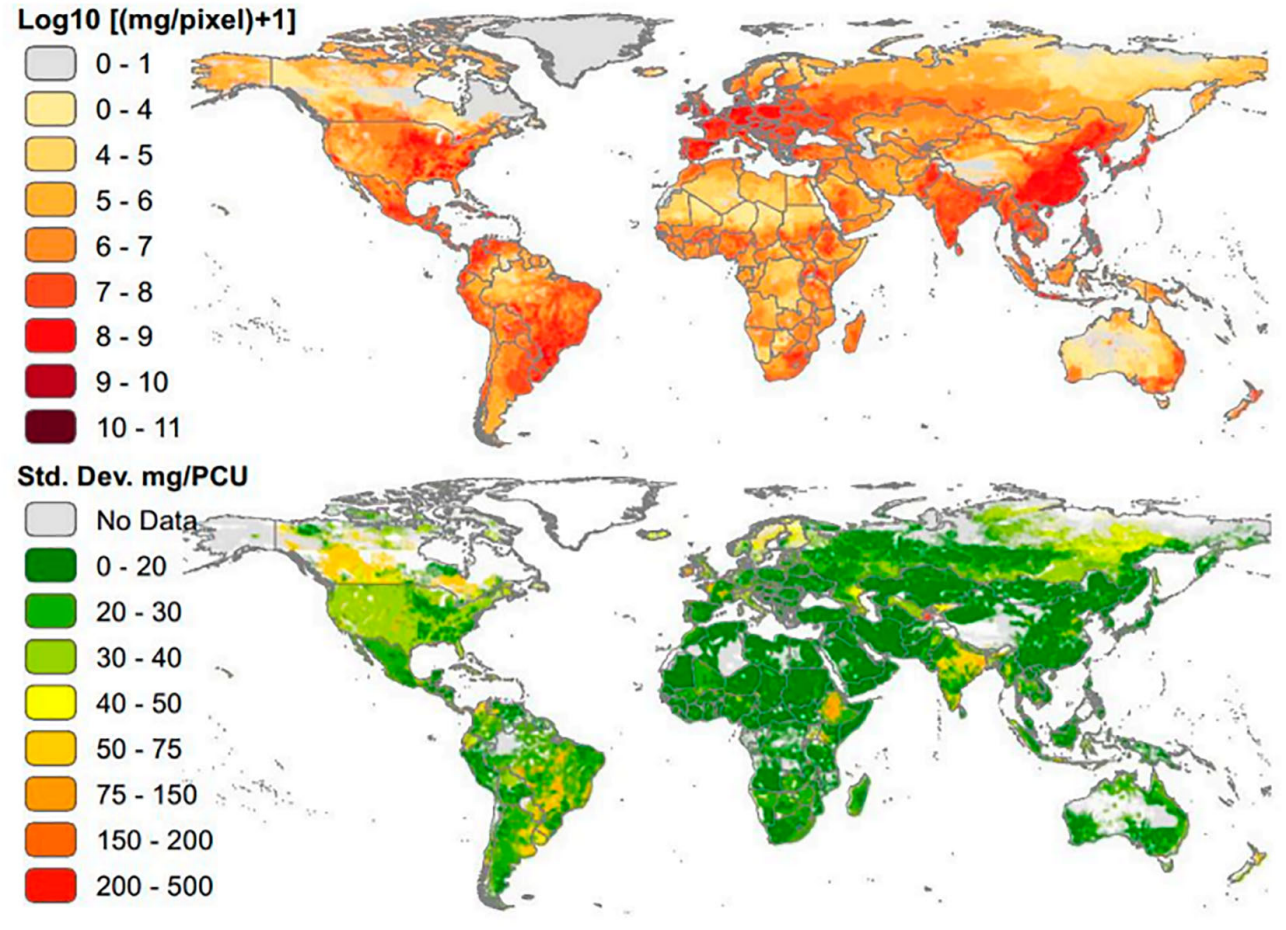
Global antimicrobial consumption in livestock in milligrams per 10 km^2^ pixels (Van Boeckel et al., 2015).

The shift toward intensive animal farming, especially in low-resource settings (**Figure 4**) (Van Boeckel et al., 2015), has led to increased AMU and heightened global risk of AMR in both humans and animals (Van Boeckel et al., 2015). A significant proportion (30–90%) of antibiotics administered to animals is excreted unchanged in urine and feces (Irfan et al., 2022), contributing to environmental contamination. Common antibiotics found in animal waste include tetracycline, flumequine, lincomycin, tylosin, oxytetracycline, doxycycline, and sulfadiazine, which are Studies have detected up to three antibiotics in pig feces and as many as eight in cow feces (Singer et al., 2016). These findings (**Figure 5**) underscore the role of livestock, especially swine, in the dissemination of AMR on farms (Udikovic-Kolic et al., 2014).

#### Sludge and manure

The antibiotics found in sludge include trimethoprim, ciprofloxacin, ofloxacin, doxycycline, sulfamethoxazole, and norfloxacin, which are less permeable to water (Clarke and Smith, 2011). The sources of influent, processing configuration, features of antibiotic splitting, and environmental factors all have a substantial impact on the levels of antibiotics in sludge and compost (Li et al., 2013). Two biocides, triclocarban and triclosan (antimicrobial, antifungal), were the most prominent analytes in an assessment of the presence of antibiotics and self-hygiene products in sewage sludge in the United States, with concentrations as high as 48.1 and 19.7 mg/kg (dry weight), respectively. The next most popular class of medications was antibiotics, which ranged in dosage from 6.8 2.3 to 0.8 0.2 mg/kg (dry weight). Ciprofloxacin, ofloxacin, 4-epitetracycline, tetracycline, minocycline, doxycycline, and azithromycin were listed in order of highest to lowest rank. Interestingly, the antibiotics and biocides that McClellan and Halden studied in 2010 (McClellan and Halden, 2010) belonged to a specific class of drugs and biocides that had a high degree of selectivity for absorption into wastewater sludge (Li and Zhang, 2010).

## Conclusion

ARGs can be transferred between bacterial populations associated with both human and livestock infection (Udikovic-Kolic et al., 2014). Although gene exchange among bacteria is theoretically possible in any environment, it most commonly occurs between closely associated bacterial species. For resistance to be successfully transmitted, both donor and recipient bacteria must coexist, at least temporarily, in the same biological niche. This implies that resistance transfer is more likely to occur among bacteria shared between humans and animals. Despite the growing recognition of AMR as a global health threat, significant knowledge gaps remain regarding the frequency, drivers, and distribution of drug-resistant pathogens in community settings. The mechanisms facilitating ARG transfer and the persistence of these genes or their bacterial hosts under varying environmental conditions are not fully understood. To address these gaps, context-specific epidemiological research is essential to explain the population-level factors contributing to the emergence and spread of AMR (Udikovic-Kolic et al., 2014). A comprehensive and cost-effective AMR response strategy must integrate a multidisciplinary approach that includes reducing antibiotic use, strengthening surveillance systems, and empowering both healthcare providers and patients to use antimicrobials prudently. Given their central role in antibiotic stewardship, healthcare professionals are critical for controlling AMR in both community and clinical settings. This study aimed to inform the development of detection, mitigation, and monitoring strategies for AMR and to support the rational use of antibiotics in treatment and management programs.
